# A novel distortion-matched anatomical imaging sequence for high-fidelity functional mapping in submillimeter-resolution fMRI

**DOI:** 10.1038/s41598-026-58377-2

**Published:** 2026-06-19

**Authors:** Seong Dae Yun, Patricia Pais-Roldán, Jeongbeen Lee, N. Jon Shah

**Affiliations:** 1 Institute of Neuroscience and Medicine 4, INM-4, Forschungszentrum Jülich, 52425 Jülich, Germany; 2 Department of Brain and Cognitive Sciences, Scranton College, Ewha Womans University, Seoul, Republic of Korea; 3 Institute of Neuroscience and Medicine 11, INM-11, JARA, Forschungszentrum Jülich, Jülich, Germany; 4 JARA - BRAIN - Translational Medicine, Aachen, Germany; 5 Department of Neurology, RWTH Aachen University, Aachen, Germany

**Keywords:** Geometric distortion correction, Distortion-matched Anatomy, EPI, FMRI, Functional mapping accuracy, MP2RAGE, Anatomy, Biological techniques, Medical research, Neuroscience

## Abstract

Echo-planar imaging (EPI), commonly employed in functional MRI (fMRI), is highly susceptible to magnetic field inhomogeneities, leading to pronounced geometric distortions in reconstructed images. These distortions can result in substantial structural discrepancies between EPI and anatomical images acquired using the magnetization-prepared 2 rapid acquisition gradient echoes (MP2RAGE) method, thereby making it challenging to achieve accurate co-registration and subsequent localization of functional mapping. This issue can be effectively addressed by employing an anatomical imaging sequence that exhibits distortion profiles identical to those in EPI, referred to here as MP2EPI (magnetization-prepared 2 EPI). While this approach enables effortless co-registration with functional scans, it also introduces geometric distortions into the anatomical reference imaging, which limits its utility for analyses that rely on morphometric measurements or atlas-based segmentation. Distortion in MP2EPI can be corrected using additional data acquired with the reversed phase-encoding (PE) direction, which, however, significantly increases total acquisition time. To overcome this limitation, this work presents a novel MP2EPI sequence that simultaneously acquires reversed PE data within a single MP2EPI acquisition, without increasing the overall scan time. The primary focus of the current work is the technical implementation and validation of this sequence in the context of submillimeter fMRI at 7T.

**Supplementary Information:**

The online version contains supplementary material available at 10.1038/s41598-026-58377-2.

## Introduction

The detection of neural activity in functional MRI (fMRI) has been explored using various imaging techniques, including conventional gradient-echo imaging^1–5^, echo-planar imaging (EPI)^6–10^, and more advanced k-space sampling approaches, such as spiral or random trajectory acquisitions^11–14^. Of these, EPI has gained widespread use due to its broad availability across MRI vendors and relatively high temporal resolution, making it particularly adept at capturing dynamic hemodynamic responses in various fMRI studies. Neural activation detected by fMRI can be directly overlaid onto functional images obtained using EPI, which, however, hinders the accurate identification of activated anatomical regions and complicates normalization into standard brain spaces such as the Montreal Neurological Institute (MNI) or Talairach coordinate systems for group-level analyses^15,16^. Instead, an additional imaging sequence usually accompanies fMRI to provide a high-resolution anatomical reference.

One commonly used method is the magnetization-prepared 2 rapid gradient echoes (MP2RAGE) sequence, offering T_1_-weighted, high-resolution anatomical images with inherent correction of B_1_ inhomogeneities through the combination of two inversion images^17,18^. The enhanced T_1_ contrast in MP2RAGE enables robust brain tissue classification among grey matter (GM), white matter (WM), and cerebrospinal fluid (CSF), facilitating comprehensive structural analyses. In particular, the accurate delineation of GM is essential for the investigation of cortical depth-dependent functional profiles in layer fMRI^19^. Alternatively, the use of T_1_-related contrast in EPI-based acquisitions has been explored in the laminar fMRI community. For instance, methods using vascular space occupancy exploit T_1_ differences between blood and tissue to generate cerebral blood volume-sensitive functional contrast, while also providing T_1_-weighted anatomical images that share the same distortion characteristics as the functional scans, thereby enabling direct registration between the two^20–22^. In parallel, a dedicated distortion-matched anatomical imaging sequence has been developed specifically for comprehensive whole-brain anatomical reference generation^23–27^. These methods also adopt the same base sequence as for fMRI, yielding nearly identical distortions, while utilizing an inversion-recovery contrast mechanism derived from EPI techniques^28–31^ designed for rapid T_1_ mapping. This approach effectively demonstrates the feasibility of generating whole-brain, T_1_-weighted anatomical contrasts within the EPI framework, and is termed here as ‘MP2EPI’ (magnetization-prepared 2 EPI). Its utility for high-resolution fMRI has been demonstrated in several previous studies, where straightforward co-registration with functional scans was achieved^19,32^.

Notwithstanding this advantage, MP2EPI also inherits the geometric distortion characteristics of EPI, making it potentially unsuitable for analyses that require undistorted anatomical fidelity, such as cortical thickness estimation, surface-based atlasing, or morphometric analyses of specific brain regions^33,34^. This limitation can be mitigated by acquiring an additional MP2EPI scan with the change of phase-encoding (PE) direction reversed, allowing for blip-up/blip-down distortion correction, as demonstrated in earlier work^24^. However, this approach requires a complete repetition of the MP2EPI acquisition, leading to a significant increase in scan time, RF energy deposition, and susceptibility to motion discrepancies between the original and reversed PE scans^35^.

To overcome the aforementioned drawback, this work aims to propose a novel MP2EPI scheme that simultaneously acquires reversed PE data within a single MP2EPI acquisition, by adapting the approach presented in earlier work^35–37^. The proposed scheme enables effective distortion correction of the MP2EPI image without requiring additional acquisition time, while ensuring effortless co-registration with the functional scans. The primary focus of the current technical report includes (i) technical implementation of the proposed sequence with appropriate protocol configuration, (ii) demonstration of the structural improvement of MP2EPI through distortion correction, and (iii) evaluation of the fidelity of functional-to-anatomical mapping based on the corrected MP2EPI, by comparing it with a brain atlas derived from a standard MP2RAGE scan, considered the gold standard. This study validates the feasibility of the proposed scheme using submillimeter-resolution (0.73 × 0.73 mm^2^) fMRI acquired on a 7 T MRI scanner.

## Materials and methods

### Proposed MP2EPI with integrated reversed PE

In MP2EPI, each inversion pulse is followed by two 3D EPI readout blocks at different inversion times (TIs), which are subsequently combined to generate a unified T_1_-weighted contrast, similar to MP2RAGE^24^. This inversion kernel is repeated multiple times to complete the required k-space encoding along the 3D EPI slice axis (typically the *z*-axis), hereafter referred to as the ‘partition’ dimension. It is important to note that 3D EPI in this context refers to an acquisition in which 2D EPI is performed for each partition, with an individual RF excitation and additional phase encoding along the *z*-axis. This readout configuration yields geometric distortion levels nearly identical to those observed in conventional 2D EPI, as commonly used in fMRI studies. This differs from 3D echo-volumar-imaging (EVI), where the entire 3D k-space is sampled following a single RF excitation, resulting in a distinct distortion behavior.

**Fig. 1:**
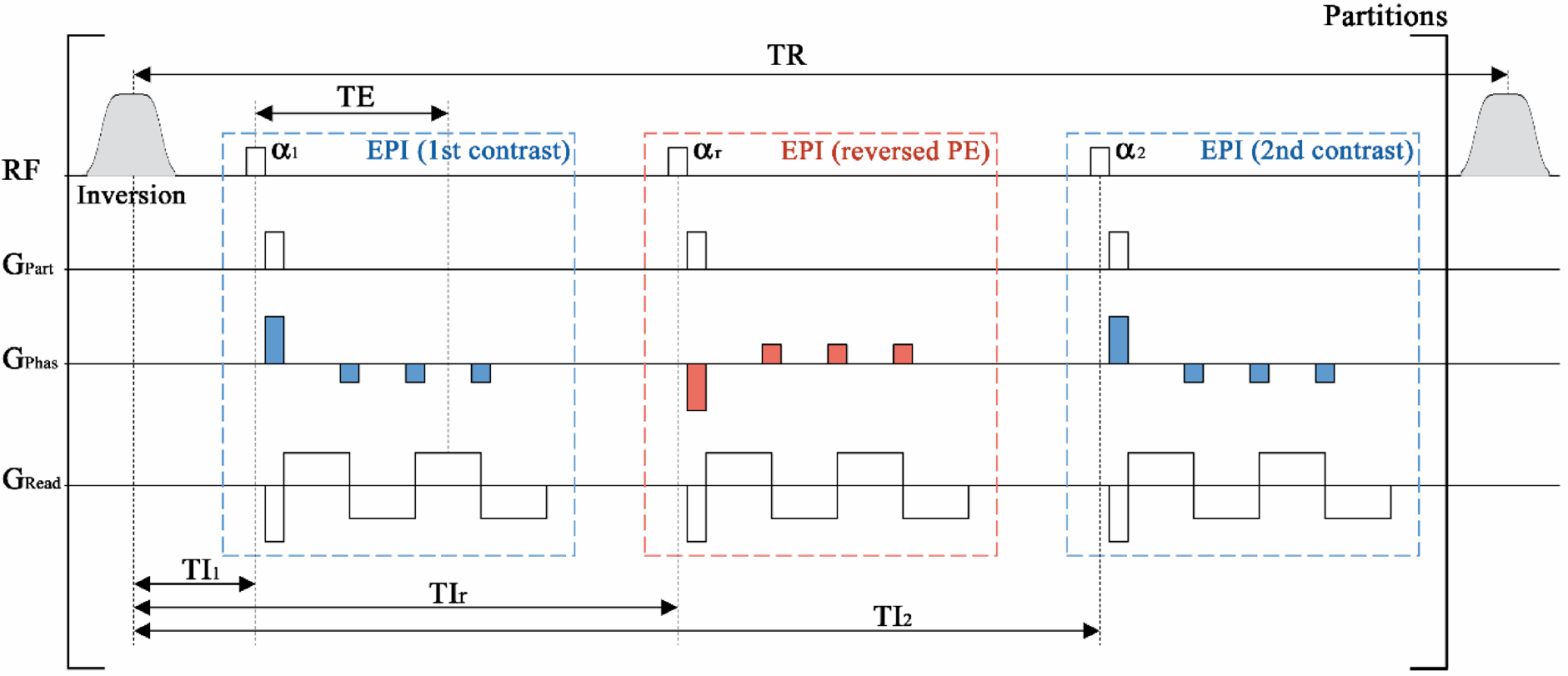
Schematic sequence diagram of the proposed MP2EPI. After inversion, 2D EPI is performed with an additional PE along the partition dimension (denoted as G_Part_). The readout configuration, determined by G_Phas_, G_Read_, ADC, and TE, was kept identical to that of the fMRI scan, ensuring identical geometric distortions. Two inversion images are acquired using the original PE direction (indicated in blue) at TI_1_ and TI_2_, resulting in a unified T_1_-contrast image, similar to standard MP2RAGE. In this scheme, an additional image is acquired using the reversed PE direction (indicated in red) at TI_r_, facilitating the blip-up/blip-down distortion correction. This imaging kernel is repeated for as many partitions as required, which, however, can be further reduced using the turbo acceleration approach (not shown here for simplicity).

Figure 1 shows a schematic diagram of the proposed MP2EPI pulse sequence that adopts the pulse sequence structure described above. The repetition of the inversion kernel can be substantially reduced by applying acceleration techniques such as parallel imaging or partial Fourier acquisition. However, to avoid potential degradation in signal-to-noise ratio (SNR) and spatial resolution along the partition dimension, these options were not implemented in the current work. As an alternative, the number of kernel repetitions was reduced by acquiring multiple 3D EPI partitions per inversion (termed turbo acceleration), with the central k-space acquired at the designated TI. In this study, a turbo acceleration factor of three was employed to prevent significant T_1_ blurring, as suggested in previous work^24^. For illustrative purposes, certain sequence components are not shown in Fig. 1, such as turbo acceleration implementation details, spoiling gradients, and navigator echoes used for EPI ghost correction.

In addition, the first and second inversion times (TIs) were configured as 1000 ms and 2700 ms, respectively, based on previous work demonstrating MP2EPI at 7T^24^. This setup introduces a substantial interval between the two TIs, allowing the incorporation of an additional contrast measurement acquired at an intermediate inversion time (TI_r_) using a reversed PE direction (see the rectangle outlined in red)^37^. Consequently, the proposed MP2EPI sequence extends the standard implementation by providing the reversed PE image for distortion correction, in addition to the conventional inversion images at TI_1_ and TI_2_, all within a single acquisition.

### Distortion correction of MP2EPI

The two inversion images (TI_1_ and TI_2_) are combined to reconstruct a unified T_1_-weighted image, which serves as the main anatomical contrast in MP2EPI. To correct for geometric distortions in this image, the reversed PE image is utilized as a reference to estimate voxel displacement using the blip-up/blip-down correction approach. In our implementation, TI_r_ for the reversed PE image is configured to yield a contrast profile, comparable to that of the unified image, ensuring that a single inversion image with appropriate tissue contrasts is sufficient for distortion correction. This strategy is motivated by previous comparative studies^38,39^, showing that while MP2RAGE offers superior quantitative contrast and B_1_ homogeneity compared to MPRAGE, the latter still provides sufficient tissue contrasts, which is presumed to be adequate for distortion correction. The TI_r_ values tested in this study were 1630 ms, 1900 ms, and 2170 ms, and their impact on the distortion correction performance was quantitatively investigated using the structural similarity index measure (SSIM)^40^.

The blip-up/blip-down method was implemented using the Advanced Normalization Tools (ANTs), a publicly available image registration and processing toolkit^41,42^. For distortion correction, the reversed-PE reference was first rigidly registered (via ‘antsRegistration’, 6 degrees of freedom) to the original MP2EPI to account for inter-scan motion. Subsequently, the pre-aligned images were registered to each other using ‘antsMultivariateTemplateConstruction2.sh’ in ANTs, which consisted of an affine alignment step followed by non-linear deformation field estimation. Specifically, the original- and reversed-PE images were iteratively deformed toward a common midpoint space using the SyN (Symmetric Normalization) transformation with a cross-correlation similarity metric. A multi-resolution optimization strategy was implemented with shrink factors of 4 × 2 × 1 and smoothing kernels (σ) of 2 × 1 × 0 voxels, respectively. The optimization was performed over a maximum of 50 × 25 × 15 iterations across the resolution scales for non-linear deformation estimation. Finally, the resulting affine matrix, deformation field and distortion-corrected midpoint template used as the spatial reference were applied to the original MP2EPI in a single resampling step via ‘antsApplyTransforms’ to obtain the final distortion-corrected MP2EPI. All resampling procedures employed Lanczos-windowed sinc interpolation to preserve high-resolution image features. Furthermore, the distortion correction results obtained from the simultaneous PE acquisition were compared with those from a conventional separate PE acquisition.

### Imaging protocols: visual fMRI

The proposed MP2EPI sequence described above was applied in a visual fMRI experiment. A standard checkerboard paradigm was designed to evoke localized activation in the visual cortex. The passive viewing task followed a simple block design, in which a black-and-white checkerboard pattern reversing at 8 Hz alternated with a low-level baseline condition (a central fixation cross). The paradigm began with four dummy scans to allow the MR signal to reach a steady state, followed by 96 fMRI volumes consisting of eight cycles of baseline and activation blocks. Each cycle comprised six volumes, resulting in a total duration of 21 s per cycle.

Functional images were acquired using a 2D gradient-echo EPI sequence with the following imaging parameters: TR/TE = 3500/22 ms, FOV (AP × LR × FH) = 210 × 210 × 117 mm^3^, matrix size = 288 × 288 with 117 axial slices (0.73 × 0.73 × 1.0 mm^3^), flip angle = 90°, partial Fourier = 5/8, parallel imaging acceleration = 3-fold, multi-band factor = 3, and receiver bandwidth = 965 Hz/Px. For anatomical reference, the proposed MP2EPI sequence was acquired using the same readout parameters as the functional scan to ensure consistent EPI distortion, except for the following: TR = 4300 ms, FOV (FH) = 192 mm, 192 axial slices, flip angles (α_1_/α_2_/α_r_) = 20°/16°/16°, parallel imaging acceleration = 3-fold along the G_Phas_ direction, averages = 3, and acquisition time = 14 min 4 s. To ensure robust parallel imaging reconstruction, the sequence acquires 3D auto-calibration signal (ACS) lines using a conventional line-by-line gradient-echo (GRE) readout (36 and 16 lines along the G_Phas_ and G_Part_ directions, respectively). Unlike EPI-based calibration, GRE-based ACS provides a more stable and PE direction-independent calibration at ultra-high fields. This enables consistent parallel imaging reconstruction for both the original and reversed PE data. An additional anatomical reference was obtained for comparison purposes using the standard MP2RAGE sequence with the following parameters: TR/TE/TI_1_/TI_2_ = 4440/2.08/840/2370 ms, FOV (FH × AP × LR) = 240 × 226 × 154 mm^3^, matrix size = 400 × 376 with 256 sagittal slices (0.6 mm isotropic), flip angles (α_1_/α_2_) = 5°/6°, parallel imaging acceleration = 3-fold, receiver bandwidth = 250 Hz/Px, and acquisition time = 10 min 47 s.

Five healthy adult participants were recruited following standard MRI safety screening procedures. All individuals were free from known medical, neurological, or psychiatric conditions. Prior to scanning, each subject received a complete explanation of the study and provided written informed consent. The study protocol, including the consent process and screening questionnaires, was approved by the institutional review board of RWTH Aachen University, Germany (approval number: EK 346/17). All experiments were performed on a 7 T Siemens Magnetom Terra scanner (Siemens Healthineers, Erlangen, Germany), equipped with a single-channel transmit and 32-channel receive head coil (Nova Medical, Wilmington, MA), as supplied by the manufacturer.

### Assessment of structural and functional fidelity

To evaluate the impact of the distortion correction, the MP2EPI scans (with and without distortion correction) were co-registered to the MP2RAGE scan, widely regarded as the anatomical gold standard, and their structural fidelity was visually assessed. In addition, the degree of structural matching between the MP2EPI scans and the MP2RAGE scan was quantitatively evaluated using *bbregister* in FreeSurfer (Martinos Center for Biomedical Imaging, MGH, Harvard Medical School, USA; https://surfer.nmr.mgh.harvard.edu/). This tool performs boundary-based registration, aligning images based on the cortical surface reconstruction and the intensity gradients at the WM-GM and GM-CSF boundaries^43^. The resultant cost function values were then compared between the uncorrected and distortion-corrected cases; the distortion-corrected case differed from the uncorrected case only in the application of the ANTs-based geometric distortion correction, whereas both datasets otherwise underwent identical processing and evaluation procedures. Specifically, this analysis was extended to investigate regional correspondence within anatomically defined regions of interest (ROIs), derived from cortical and subcortical parcellations generated by FreeSurfer.

For functional analysis, after pre-processing the fMRI data, including realignment, co-registration to the original uncorrected MP2EPI, and spatial smoothing with a Gaussian kernel width of 0.73 mm, the first-level analysis was performed using the generalized linear model in SPM12 (Wellcome Department of Imaging Neuroscience, UCL, UK; https://www.fil.ion.ucl.ac.uk/spm/software/spm12/). To evaluate the fidelity of functional mapping onto the MP2EPI scans, the resulting activated voxels were overlaid on both the uncorrected and distortion-corrected MP2EPI images. Specifically, to align the functional results with the distortion-corrected MP2EPI, the original activation maps were transformed using the same warping field applied for the distortion correction of the MP2EPI image. Here, GM boundaries extracted from the MP2RAGE image were additionally overlaid to examine the spatial correspondence between the functional activations and the anatomical reference as defined by MP2RAGE.

## Results

### Reconstructed images: 2D EPI and MP2EPI

**Fig. 2:**
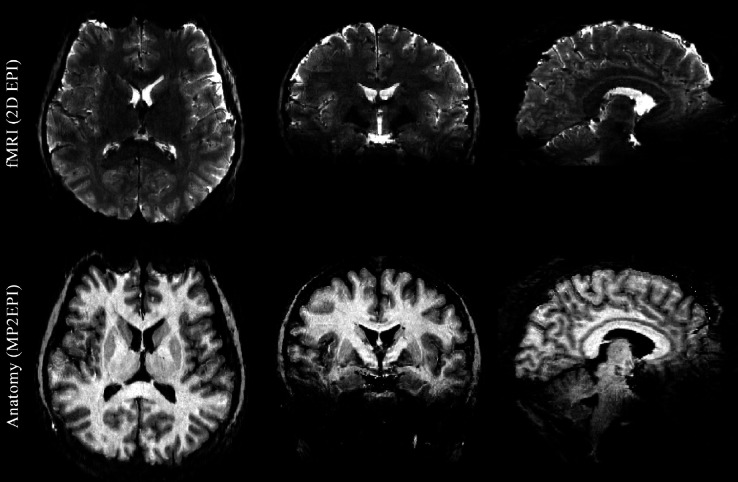
Reconstructed fMRI and MP2EPI scans. Images presented in three orientations show nearly identical geometric distortions in the functional (top) and anatomical (bottom) scans acquired using the 2D EPI and proposed MP2EPI sequences, respectively.

Figure 2 presents reconstructed images from functional and anatomical scans acquired using 2D EPI and MP2EPI, respectively, at the same anatomical location. The MP2EPI images demonstrate a high degree of similarity to the 2D-EPI images in terms of anatomical geometry, including distortion patterns, as evident in the axial, coronal, and sagittal views. This suggests that MP2EPI preserves the same distortion characteristics as 2D EPI, thereby enabling accurate co-localization between the two. The main difference between the images lies in their contrast; 2D EPI exhibits T_2_^*^-weighted contrast, whereas MP2EPI shows T_1_-weighted contrast.

### Distortion correction: MP2EPI

**Fig. 3:**
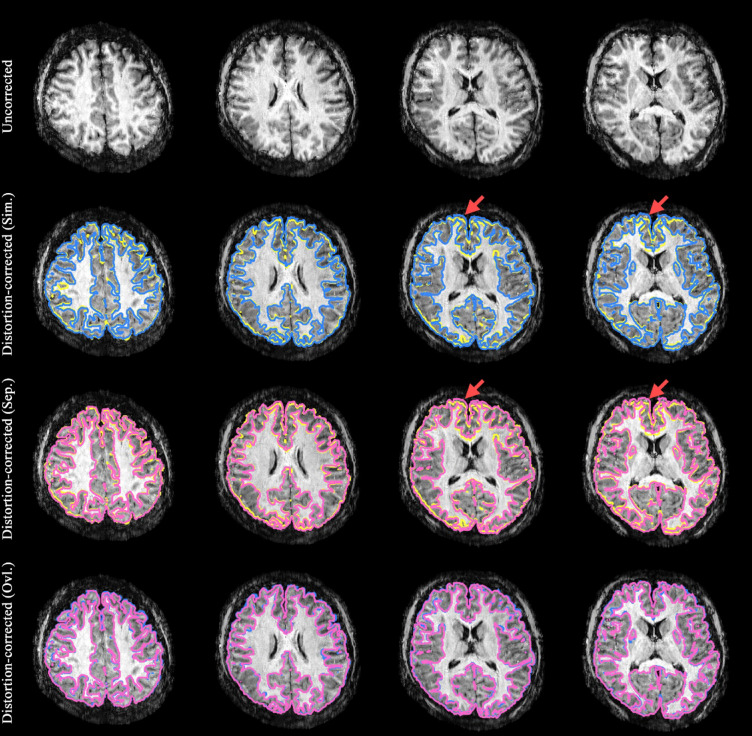
Results of blip-up/blip-down distortion correction. The top row shows the original MP2EPI images, while the second and third rows present distortion-corrected results using simultaneously and separately acquired reversed PE data, respectively. GM and WM boundaries are overlaid for visual comparison, with original boundaries in yellow and corrected boundaries in blue (simultaneous PE) and magenta (separate PE). A substantial reduction in geometric distortion is particularly evident in the frontal lobe (red arrows). The bottom row displays an overlay of the corrected boundaries from both approaches, demonstrating close agreement between the two approaches.

Figure 3 presents original, uncorrected MP2EPI images at four representative axial slice locations (top row), in comparison to the corresponding distortion-corrected images (second row) obtained using the simultaneously acquired reversed PE images at a TI_r_ value of 1630 ms (denoted as TI_r1_). For comparison, distortion-corrected results obtained from separately acquired PE data are also provided (third row). When compared to the uncorrected images, both distortion-corrected versions exhibit a substantial reduction in geometric distortions, particularly in the frontal lobe as highlighted by the red arrows in both results (second and third rows). To facilitate visual assessment of distortion correction, GM and WM boundaries were extracted and overlaid; the original distorted boundaries are indicated in yellow, while the corrected boundaries are shown in blue (simultaneous PE) and magenta (separate PE). The bottom row presents an overlay of both corrected boundaries, demonstrating a high degree of spatial agreement between the two approaches and indicating that the simultaneous acquisition provides a comparable correction performance to the conventional separate scan.

**Fig. 4:**
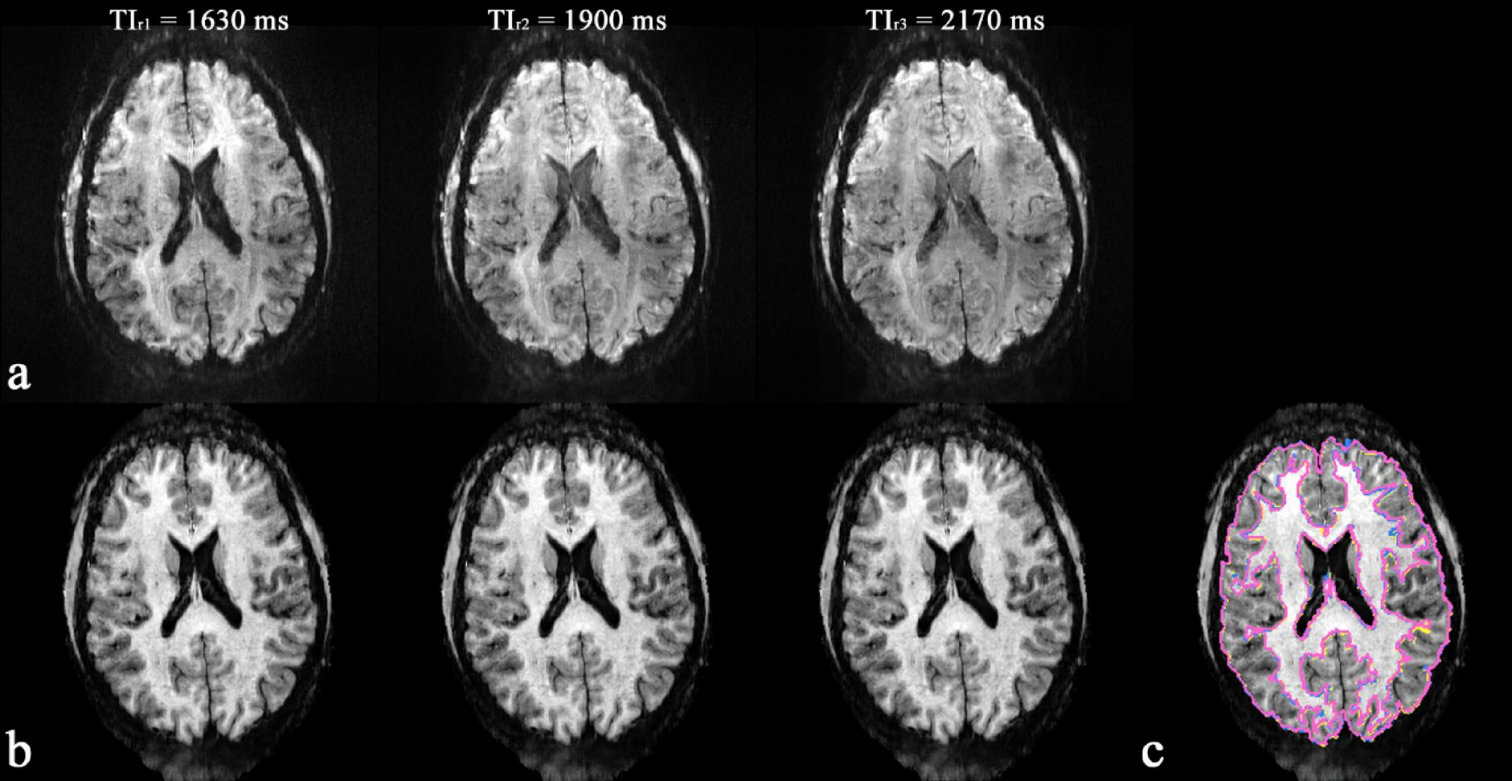
Comparison of distortion-corrected MP2EPI at different TI_r_ values. (**a**) The reversed PE images acquired at TI_r_ values of 1630 ms (TI_r1_), 1900 ms (TI_r2_), and 2170 ms (TI_r3_), respectively. (**b**) The corresponding distortion-corrected MP2EPI images. (**c**) The boundaries of the GM, WM, and ventricles extracted from each distortion-corrected image, overlaid on the distortion-corrected image from TI_r1_. The boundaries obtained from TI_r1_, TI_r2_, and TI_r3_ are shown in yellow, blue, and magenta, respectively. Visual inspection of the reconstructed images and boundaries suggests that the three distortion-corrected images have features highly comparable to each other.

Figure 4 shows distortion correction results obtained using reversed PE images acquired at alternative TI_r_ values (1900 ms and 2170 ms, denoted as TI_r2_ and TI_r3_, respectively), alongside the TI_r1_ results for direct visual comparison; the corresponding reversed PE images used for distortion correction are also shown. Although the tissue contrast in the reversed PE image at TI_r1_ is not as pronounced as that in the combined MP2EPI image, it still provides sufficient distinction between GM, WM, and CSF, comparable to the MP2EPI image, thereby enabling effective distortion correction. For TI_r2_ and TI_r3_, the tissue contrast becomes less distinct, with increased CSF signal intensity and diminished contrast between GM and WM. Nonetheless, distortion correction using these images yielded results comparable in quality to those obtained at TI_r1_, as shown in Fig. 4b, indicating that the correction process remained effective despite the reduced tissue contrast. This can be clearly verified in Fig. 4c, through the comparison of the boundaries of the GM, WM, and ventricles extracted from each distortion-corrected MP2EPI image.

**Fig. 5:**
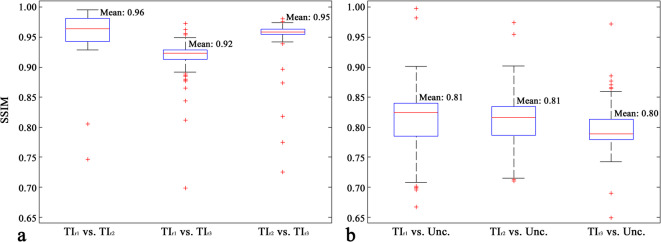
Performance assessment of distortion correction using SSIM. (**a**) Pairwise comparison of distortion-corrected MP2EPI images (TI_r1_ vs. TI_r2_, TI_r1_ vs. TI_r3_, and TI_r2_ vs. TI_r3_), demonstrating highly comparable structural similarity between the three corrected datasets. (**b**) Comparison of uncorrected and distortion-corrected MP2EPI images for the three TIr values, showing significant geometric differences between uncorrected and corrected images, with a consistent degree of structural deviation across all TIr values. The mean value is also indicated in each boxplot.

The structural similarity between the distortion-corrected MP2EPI images obtained from different TI_r_ values was further quantified using SSIM. Specifically, slice-wise SSIM values were computed for three pairwise comparisons: TI_r1_ vs. TI_r2_, TI_r1_ vs. TI_r3_, and TI_r2_ vs. TI_r3_. The results are summarized as box plots in Fig. 5a. For the first comparison (see the leftmost box plot), the SSIM values exhibited a median above 0.95 with a narrow interquartile range, outlined by the blue box, and both the first (Q1) and third (Q3) quartiles were tightly clustered between 0.95 and 1.0, reflecting a very high structural correspondence between the two images. Similarly, results for other comparison sets (see the middle and rightmost box plots) also yielded consistently high SSIM values. The mean SSIM values for the three comparisons were 0.96, 0.92, and 0.95, respectively. These findings indicate that the distortion correction process was highly robust across the tested TI_r_ values, showing minimal sensitivity to the values employed here. To complement these findings, each distortion-corrected image was also compared with the uncorrected MP2EPI image using SSIM (see Fig. 5b). The resulting mean SSIM values were 0.81, 0.81, and 0.80 for TI_r1_, TI_r2_, and TI_r3_, respectively, indicating considerable geometric differences between the uncorrected and corrected images. Furthermore, the similarity in SSIM across the three TI_r_ values suggests that a consistent degree of structural deviation was introduced by the distortion correction process.

### Structural fidelity

**Fig. 6:**
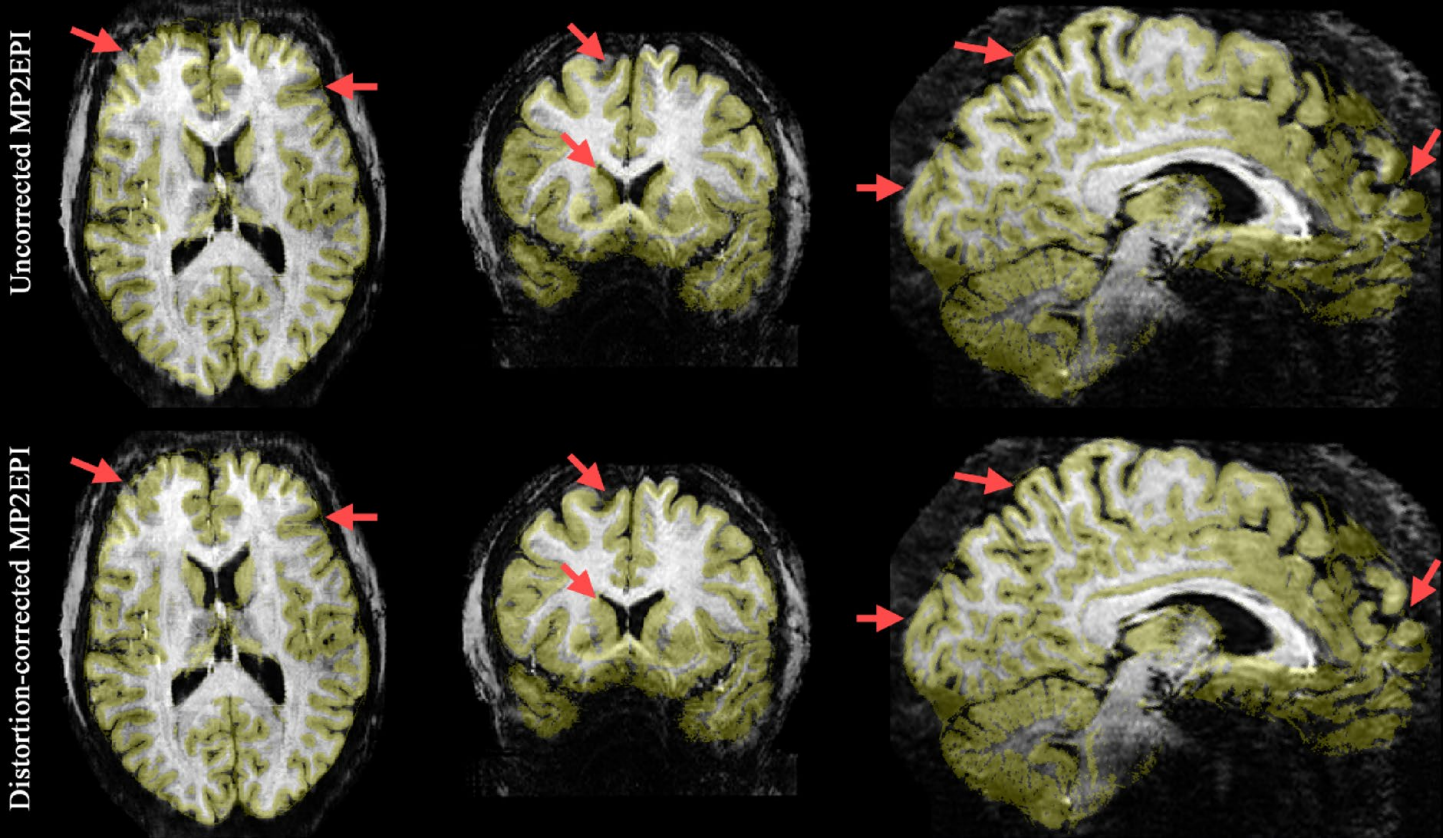
Visual assessment of co-registration to MP2RAGE. The top and bottom rows show the uncorrected and distortion-corrected MP2EPI scans, respectively, each co-registered to the MP2RAGE scan. GM regions extracted from the MP2RAGE scan (yellow) are overlaid to highlight the substantial improvement in co-registration achieved through distortion-correction. This is particularly evident in the ventricular, frontal, and occipital regions, as indicated by the red arrows.

The improvement in structural fidelity following distortion correction is illustrated in Fig. 6, which shows uncorrected and distortion-corrected MP2EPI images in axial, coronal, and sagittal views, overlaid with GM masks extracted from the MP2RAGE scan (highlighted in yellow). In the uncorrected images, substantial misalignment is observed between the MP2EPI and MP2RAGE-derived GM regions, with background and ventricular voxels from MP2EPI erroneously included within the GM areas. In addition, cortical voxels around the frontal and occipital lobes are found to be partially excluded from the GM regions (see areas indicated by the red arrows).

**Fig. 7:**
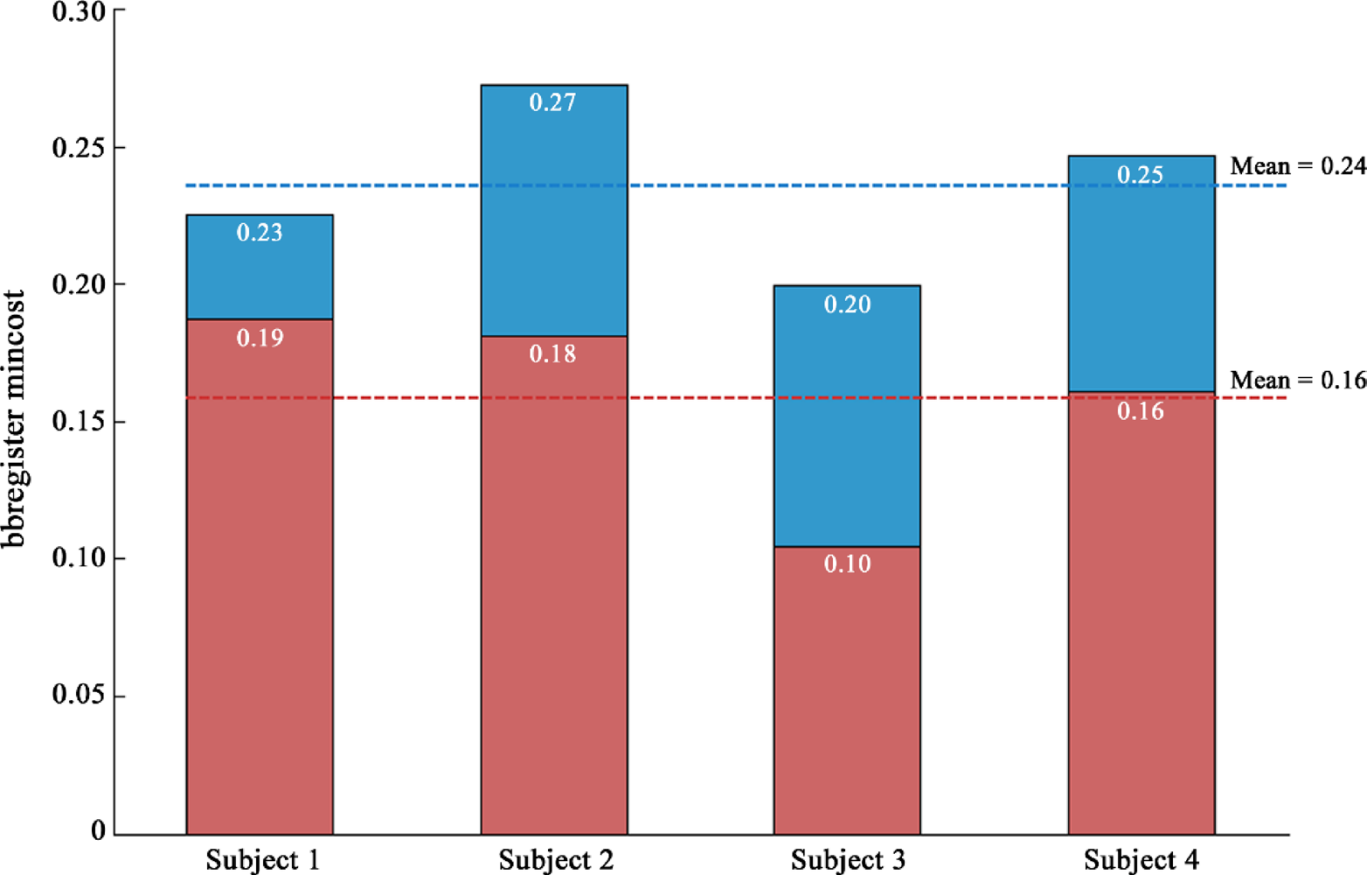
Results of the boundary-based registration cost function. Each bar shows the ‘mincost’ values obtained for the uncorrected (blue) and distortion-corrected (red) MP2EPI scans. A substantial reduction in mincost is consistently observed across all subjects, indicating improved co-registration accuracy through distortion correction.

This misalignment is significantly reduced in the distortion-corrected case, where the overlaid GM masks more accurately conform to the anatomical boundaries defined by the MP2EPI, indicating improved structural correspondence between distortion-corrected MP2EPI and MP2RAGE. Figure 7 shows the results of the boundary-based registration cost function values (‘mincost’), obtained for the uncorrected and distortion-corrected MP2EPI scans. Across all four subjects, the mincost values were substantially reduced (mean decrease from 0.24 to 0.16), indicating that the distortion correction led to enhanced anatomical alignment with the MP2RAGE reference.

**Fig. 8:**
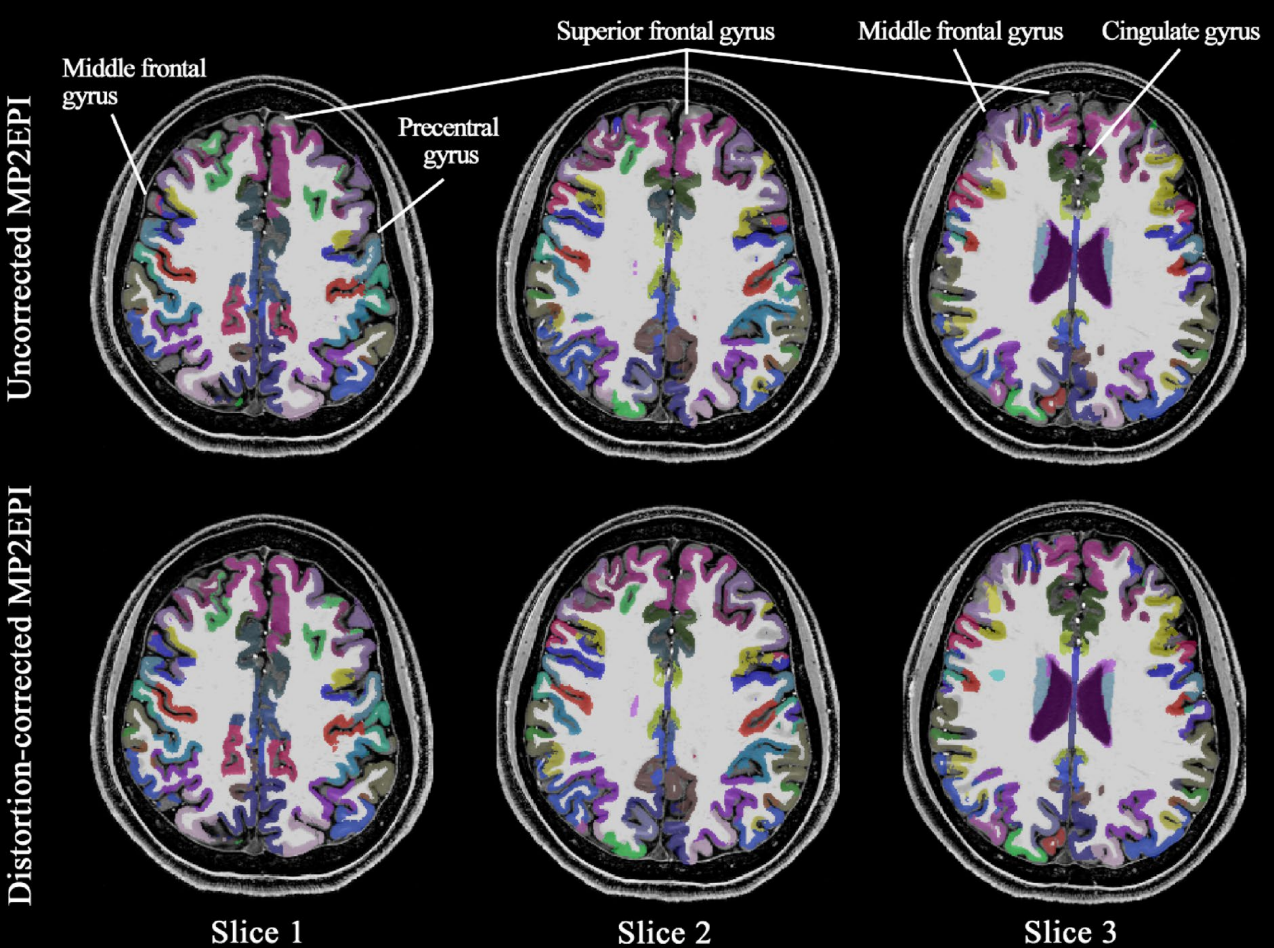
Results of the anatomical ROI parcellation. The parcellated cortical ROIs, obtained from uncorrected (top) and distortion-corrected (bottom) MP2EPI scans, are overlaid on the MP2RAGE scan. The anatomical ROIs, shown in distinct colors, are more accurately aligned with the MP2RAGE scan in the distortion-corrected case when compared to the uncorrected one. This is particularly evident in the indicated regions: the superior frontal, middle frontal, precentral, and cingulate gyri.

This improved structural fidelity was further confirmed by the anatomical ROI analysis performed using FreeSurfer. Figure 8 displays cortical parcellation results obtained from both uncorrected and distortion-corrected MP2EPI, overlaid onto the MP2RAGE image. When compared to the uncorrected case, the distortion-corrected results demonstrate that anatomical ROIs such as superior frontal, middle-frontal, precentral, and cingulate gyri are more accurately aligned with the underlying MP2RAGE anatomy, supporting the enhanced spatial correspondence achieved through distortion correction.

### Functional fidelity

**Fig. 9:**
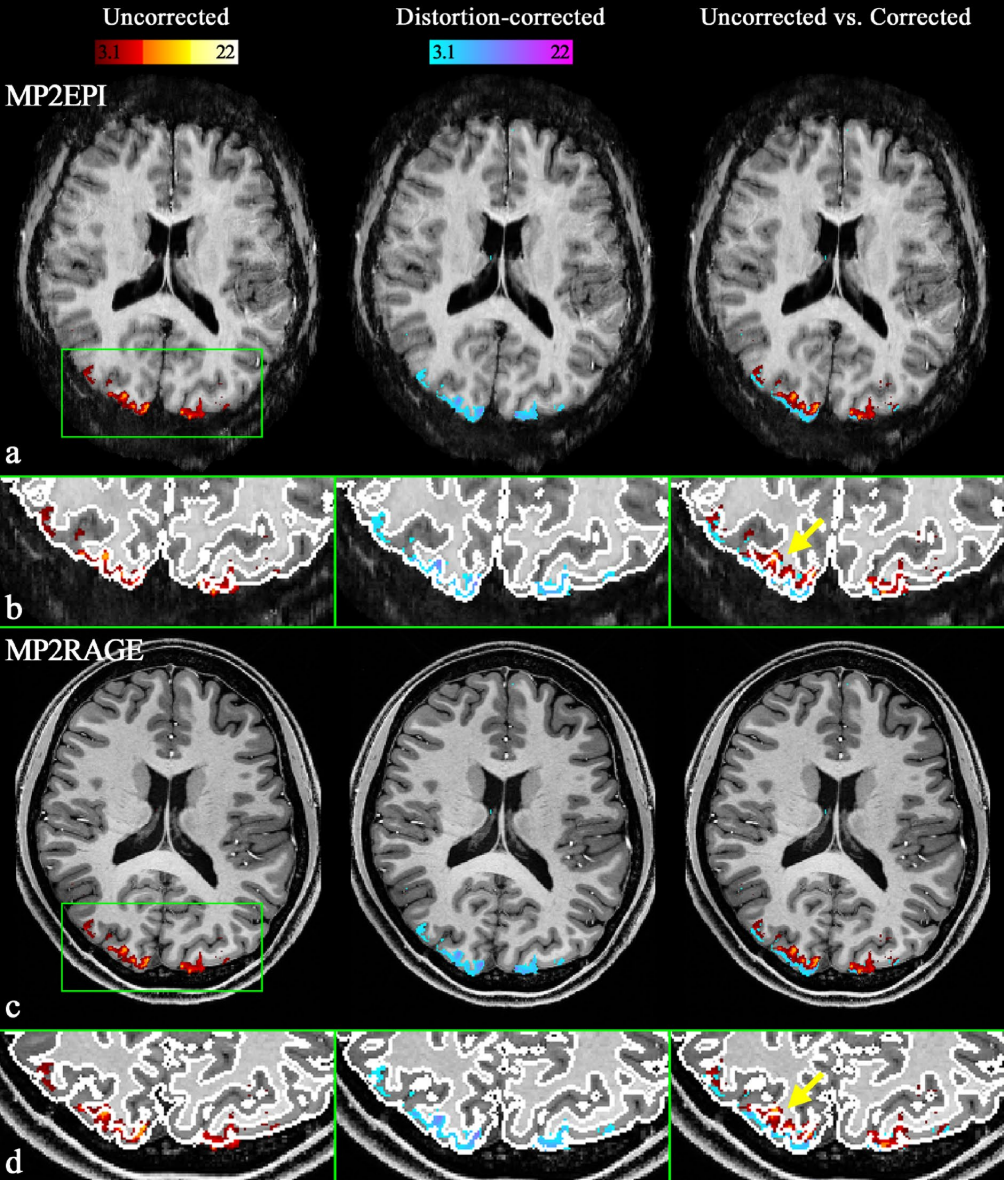
Results of visual fMRI on MP2EPI and MP2RAGE. (**a**) Activated voxels mapped onto the uncorrected (left) and distortion-corrected MP2EPI images (middle), with both results overlaid on the distortion-corrected MP2EPI (right). (**b**) Enlarged view of the green rectangular ROI, with GM boundaries (white) extracted from the corresponding MP2EPI image in panel (a), highlighting differences in the localization of activated voxels between the uncorrected and distortion-corrected cases. The same panel organization was applied for MP2RAGE in panels (**c**) and (**d**), demonstrating that the activated voxels after distortion correction showed much closer spatial correspondence to the GM derived from MP2RAGE, compared to the uncorrected case.

Figure 9a displays activated voxels at a representative slice location, mapped on the uncorrected MP2EPI and distortion-corrected MP2EPI images. In the last column, activated voxels from both cases were overlaid, clearly revealing the spatial mismatch between the two. Figure 9b presents an enlarged view of the green rectangular ROI for both the uncorrected and distortion-corrected MP2EPI images, with the GM boundaries extracted from each case outlined in white. This image panel demonstrates that in both cases, the activated voxels are well aligned with their respective GM boundaries, indicating that the co-registration between the functional and MP2EPI scans was effective, thereby ensuring consistent mapping within the structural space of the MP2EPI scans.

However, when the activated voxels were mapped on the co-registered MP2RAGE scan (Figs. 9c and d), the activation map from the uncorrected MP2EPI showed substantial misalignment relative to the MP2RAGE-derived GM highlighted in yellow. In contrast, the distortion-corrected MP2EPI showed a much closer spatial correspondence with the MP2RAGE GM, suggesting that distortion correction effectively enhances structural fidelity to a level comparable to that of the MP2RAGE. The practical benefit of this improved spatial fidelity is further illustrated by cortical line profile analysis (Supplementary Fig. S1). The results demonstrate that distortion correction in MP2EPI enhances alignment with the MP2RAGE, thereby providing a more accurate anatomical reference for reliable layer-specific functional mapping.

## Discussion

This concise technical report demonstrates the implementation of a novel MP2EPI scheme that enables the simultaneous acquisition of reversed phase-encoding (PE) data within a single scan. This approach eliminates the need for additional MP2EPI acquisitions with reversed PE direction, thereby reducing total scan time and minimizing cumulative RF energy deposition. A previous approach^44^ proposed a scheme that utilizes multiple shots to acquire blip-up and blip-down EPI data for diffusion MRI. This method uses multiple shots to sample complementary subsets of k-space with opposite PE directions, requiring a model-based joint reconstruction to recover a distortion-free image. In contrast, our proposed MP2EPI sequence acquires fully sampled blip-up and blip-down images at every excitation (i.e., per partition in the 3D EPI framework) with dedicated TIs. This scheme facilitates the use of standard reconstruction pipelines, thereby enabling the direct application of conventional post-processing methods for distortion correction.

When compared to MP2RAGE, which employs a conventional 3D gradient-echo acquisition for readout, MP2EPI exhibits relatively lower SNR due to the inherent characteristics of the EPI readout. This is primarily attributed to prolonged echo trains leading to substantial T_2_ decay, increased thermal noise from high receiver bandwidth, and phase inconsistencies between PE lines that result in destructive interference, issues that are challenging to entirely eliminate in practice. To mitigate the low SNR, three signal averages were used in the current protocol, building upon a similar strategy adopted in previous work^24^. This contributed to the total acquisition time of approximately 14 min. Therefore, given the requirement of a relatively long acquisition time for MP2EPI, the proposed simultaneous acquisition of reversed PE data provides a substantial advantage in the acquisition of reversed PE data. In this initial implementation, no parallel imaging acceleration or partial Fourier technique was applied along the 3D EPI partition direction to avoid further SNR degradation or spatial blurring, which could complicate interpretation in this first demonstration. Our implementation also incorporates a conventional GRE readout for the 3D ACS acquisition. Compared to alternative EPI-based calibration frameworks such as FLEET, a line-by-line GRE-based calibration is free from T_2_^*^-induced blurring and EPI-specific geometric distortions. This advantage is particularly pronounced in high-resolution, low-bandwidth fMRI protocols. Although the GRE-based strategy entails an increased acquisition time (14.4 s in our protocol), it provides a more stable, PE-direction-independent calibration kernel for parallel imaging reconstruction. This ultimately facilitates consistent and robust reconstruction across both the original and reversed-PE MP2EPI data. Future studies may explore optimized protocols incorporating acceleration to further reduce scan time, which is beyond the scope of the current study.

The primary scope of the present work is the implementation of the proposed simultaneous acquisition of reversed-PE MP2EPI within a single scan, thereby enhancing anatomical fidelity through geometric distortion correction without requiring external calibration scans. In the current implementation, the reversed-PE reference was acquired using the same GE-EPI readout as the original MP2EPI acquisition, differing only in the PE direction, a strategy routinely adopted in the community. However, GE-EPI readouts are susceptible to T_2_^*^-induced signal dropouts in regions of high susceptibility, which may confound local distortion estimation. The proposed framework could, in principle, benefit from alternative reference strategies, such as integrating an SE-EPI-based reversed-PE block as explored in the prior literature^45^. However, careful consideration would be required regarding the increased SAR associated with refocusing pulses, as well as potential cross-contrast effects (i.e., SE-to-GE) which may influence field estimation performance^45^.

Geometric distortion in MP2EPI was substantially reduced using the reversed PE data acquired with the proposed scheme. The reversed PE image at TI_r1_ exhibited nearly identical image contrast to that of the unified T_1_ image obtained with the original PE direction. Nevertheless, the performance of distortion correction was highly comparable across different TI_r_ values tested in this study, as verified by the visual inspection and SSIM analysis. This robustness can be attributed to the fact that warping field estimation during distortion correction relies on advanced image registration, which focuses on aligning anatomical structures. As a result, the method remained effective even in the presence of substantial signal intensity differences between the reversed and original PE images. These distortion-corrected images showed substantially improved structural alignment with the MP2RAGE scan, demonstrating the effectiveness of distortion correction using the proposed scheme. This improvement was further supported by the anatomical ROI parcellation of cortical regions, showing that the misalignments observed in the uncorrected MP2EPI were significantly reduced in the distortion correction results.

Crucially, the enhancement in structural fidelity directly translated to improved functional mapping accuracy. In the uncorrected case, activated voxels were well localized within the cortical areas defined by MP2EPI, reflecting effective co-registration between the functional scans and the anatomical reference derived from using 2D EPI and uncorrected MP2EPI, respectively. However, these activation patterns showed substantial mismatch when compared to the cortical boundaries defined by MP2RAGE, due to geometric distortions present in both the functional and anatomical scans. In contrast, for the distortion-corrected MP2EPI, the activation maps that were also corrected using the same warping field were well aligned with the cortical ribbon defined by both the corrected MP2EPI and the MP2RAGE. This demonstrates the impact of distortion correction on the improvement of the spatial fidelity in functional mapping.

While the structural fidelity of MP2EPI was enhanced by distortion correction, the sequence remains susceptible to signal dropout near air-tissue boundaries, a common issue in EPI images caused by susceptibility differences. To mitigate this limitation, various strategies have been attempted, including multi-echo acquisitions^46,47^, tailored RF pulse designed with a hyperbolic secant or quadratic phase profile^48–50^, and deep learning techniques^51^. These methods have demonstrated an effective reduction of signal dropout, leading to the improved detectability of functional signals. A similar strategy could be incorporated into our MP2EPI framework in future studies, potentially yielding anatomical structures that more closely resemble those obtained with MP2RAGE. In particular, deep learning techniques have also been shown to improve SNR^52^, which may enable significant reductions in acquisition time by decreasing the number of signal averages required in the present protocol. Despite this limitation, the feasibility of using MP2EPI for submillimeter fMRI was demonstrated in the context of a visual task, showing enhanced structural and functional fidelity for the distortion-corrected MP2EPI. The standard automatic cortical parcellation provided by FreeSurfer successfully identified most cortical regions in both the uncorrected and distortion-corrected MP2EPI scans.

In conclusion, this work presents a novel MP2EPI scheme that enables the simultaneous acquisition of reversed PE data within a single measurement. This scheme facilitates blip-up/blip-down distortion correction without requiring additional MP2EPI acquisitions solely for the purpose of reversing the PE direction, thereby improving efficiency in acquisition time and reducing RF energy deposition to subjects. The enhancement in structural fidelity through distortion correction was verified by direct comparison with the MP2RAGE scan. The utility of the proposed MP2EPI approach was demonstrated in the context of submillimeter-resolution (0.73 × 0.73 mm^2^) fMRI at 7T. Both the uncorrected and corrected MP2EPI images provided anatomical reference with distortion profiles matched to those of the corresponding functional scans, enabling faithful co-registration and functional mapping. However, in the distortion-corrected case, the activated voxels exhibited substantially improved alignment with the cortical regions defined by MP2RAGE, reflecting enhanced structural fidelity and functional mapping accuracy achieved by distortion-corrected MP2EPI.

## Supplementary Information

Below is the link to the electronic supplementary material.

Supplementary Material 1

## Data Availability

This study presents human in vivo brain data, the sharing of which may raise concerns regarding the protection of personal data and privacy. The Council of Europe’s policy on data protection specifically applies to health-related data (CM/Rec(2019)2), and ethical guidelines and internal administrative documents further ensure the confidentiality of in vivo data and any metadata derived from the original data. Data sharing is available upon request to Prof. N. Jon Shah (n.j.shah@fz-juelich.de) under a formal data-sharing agreement. Sharing is contingent on the consent of the subjects whose data are involved, and they will be informed in advance. The custom scripts used for image processing and analysis in this study are publicly available at: https://github.com/SeongDaeYun/NovelMP2EPI.
