# Supplementary material for: A novel distortion-matched anatomical imaging sequence for high-fidelity functional mapping in submillimeter-resolution fMRI
Source: Sci Rep. 2026 Jun 19;16:19182. doi: 10.1038/s41598-026-58377-2 (PMC13282486; doi:10.1038/s41598-026-58377-2)
Supplement: Supplementary file 1 — Supplementary Material 1 [file 41598_2026_58377_MOESM1_ESM.docx]

**Supplementary Information**

**A Novel Distortion-Matched Anatomical Imaging Sequence for High-Fidelity Functional Mapping in Submillimeter-Resolution fMRI**

Seong Dae Yun^*^, Patricia Pais-Roldán, Jeongbeen Lee, and N. Jon Shah

*** Correspondence**:

Dr. Seong Dae Yun*

Institute of Neuroscience and Medicine 4, Forschungszentrum Jülich, 52425 Jülich, Germany

[s.yun@fz-juelich.de](mailto:s.yun@fz-juelich.de)

**Impact of distortion correction on MP2EPI as an anatomical reference**

As demonstrated in the main text, the distortion-corrected MP2EPI images show improved alignment with the MP2RAGE reference compared to uncorrected MP2EPI. This alignment discrepancy is further illustrated in Supplementary Figure S1 (panels a and b), where GM boundaries derived from uncorrected (blue) and distortion-corrected (red) MP2EPI are overlaid on the MP2RAGE.

**
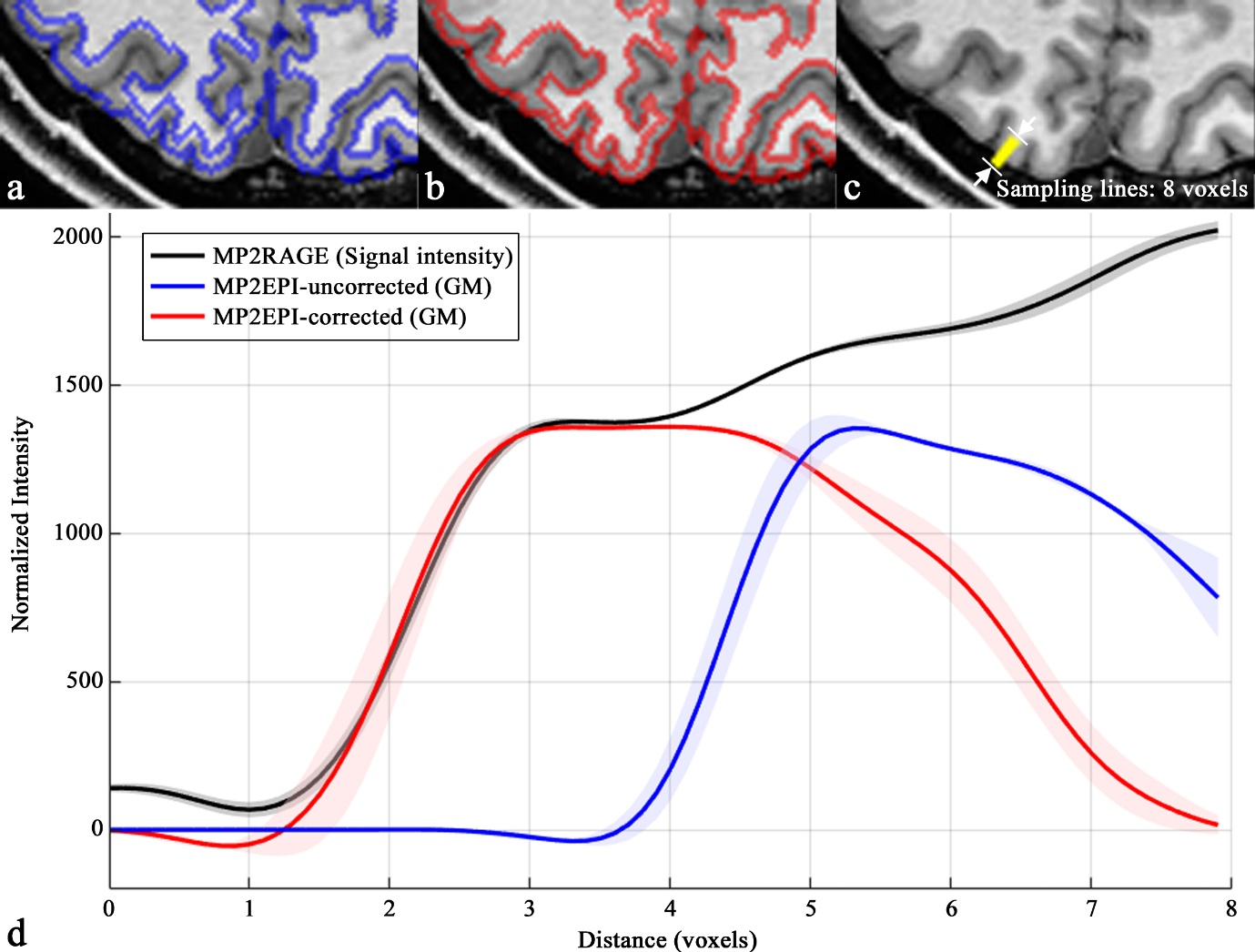
**

**Supplementary Figure S1. Cortical profiling and spatial alignment of MP2EPI.** GM boundaries extracted from (**a**) uncorrected and (**b**) distortion-corrected MP2EPI, overlaid on MP2RAGE. (**c**) Ten line trajectories (each spanning 8 voxels) defined for cortical depth-dependent signal profiling. (**d**) Signal intensity profiles from MP2RAGE (black), alongside intensity-normalized GM probability profiles from uncorrected (blue) and corrected (red) MP2EPI, demonstrating that distortion correction substantially enhances spatial alignment with MPRAGE within GM regions.

The improved alignment facilitates more accurate delineation of GM regions and suggests a more reliable basis for cortical depth-dependent functional analysis. As shown in panel c, ten sampling lines (yellow), each spanning 8 voxels across the cortical ribbon, were defined. The corresponding mean ± standard deviation (STD) intensity profiles from the MP2RAGE were computed along these trajectories (black, panel d). GM probability profiles extracted from both uncorrected and distortion-corrected MP2EPI were overlaid for comparison. The results indicate that the corrected MP2EPI more closely matches the MP2RAGE reference, supporting enhanced accuracy in cortical boundary definition, whereas the uncorrected MP2EPI exhibits substantial spatial mismatch.
